# The relationship between fear of missing out and mobile phone addiction among college students: the mediating role of depression and the moderating role of loneliness

**DOI:** 10.3389/fpubh.2024.1374522

**Published:** 2024-03-22

**Authors:** Nana Liu, Siyu Zhu, Weixin Zhang, Yanyan Sun, Xinyao Zhang

**Affiliations:** Department of Social Medicine and Health Management, School of Public Health, Jilin University, Changchun, China

**Keywords:** fear of missing out, depression, loneliness, mobile phone addiction, college students

## Abstract

**Background:**

Mobile phone addiction has adverse influences on the physical and mental health of college students. However, few studies shed light on the effect of fear of missing out on mobile phone addiction and the underlying mechanisms among college students.

**Methods:**

To explore their associations, the present study used the Fear of Missing Out Scales (FoMOS), Loneliness Scale (USL-8), Mobile Phone Addiction Index Scale (MPAI), and Depression-Anxiety-Stress Questionnaire (DASS-21) to investigate 750 college students.

**Results:**

The results suggested that fear of missing out significantly positively predicted mobile phone addiction. This direct effect could be mediated by depression, and the indirect effect of fear of missing out on mobile phone addiction could be moderated by loneliness. Specifically, the indirect effect was stronger for students with high levels of loneliness.

**Conclusion:**

This study provides a theoretical basis for developing future interventions for mobile phone addiction in higher education students.

## 1 Introduction

In the era of information explosion, the mobile Internet has dramatically changed the public’s habits of mind, affected individual behaviors and psychosocial resilience. Meanwhile, smartphones have become a significant media tool for reshaping human existence and interpersonal interactions. As of June 2023, the number of Chinese Internet users reached 1.079 billion, and the percent of Internet penetration was 76.4%. The proportion of consumers using mobile phones to access the Internet reached 99.8% (1). Although smartphone is conducive to communicating online, shopping online, looking for entertainment, studying, and so on, which brings great convenience to their studies and lives, excessive use of mobile phones may affect the physical and mental health of individuals. Numbers of students perceive it tough to control the frequency and time of mobile phone use, which invisibly triggers a psychological disease called mobile phone addiction to some extent (2). Mobile phone addiction is also thought as mobile phone dependence, mobile phone abuse, problematic mobile phone use, or mobile phone syndrome. Bianchi and Phillips consider mobile phone addiction to be a behavioral addiction, manifested by the presence of craving symptoms, withdrawal symptoms, tolerance, escape from other problems, and negative life consequences (3). Lapierre believes that mobile phone addiction is an inability to autonomously control and excessive use of mobile phones, which adversely affects individuals’ daily life (4). A meta-analysis showed that the detection rate of mobile phone addiction was 23.0% in the college student population (5).

Previous studies have shown that college students with mobile phone addiction suffer from problems such as poor sleep quality, interpersonal tension, and decreased efficiency in both study and work (5–7). Excessive immersion in mobile phone use would also further affect their stress coping and self-control ability (8–10). In addition, inattention plays a negative role in concentrating on the lessons, which leads to weakening the initiative and continuity of learning, increasing the psychological risk of anorexia, and even bringing passive emotions such as anxiety and depression among college students (11). In conclusion, mobile phone addiction has become a primary public health problem among college students, it also has been thought as a hot issue in the related research field. At the same time, college students prove a vital reserve for social development, not only is the level of physical and mental health closely associated with their own and family circumstances but also with the future development of the whole country’s society. Therefore, this study chooses college students as the research object to better analyze the mechanism and influencing factors of college students’ mobile phone addiction.

### 1.1 The relationship between fear of missing out and mobile phone addiction

Fear of Missing Out (FoMO), is defined as a diffuse anxiety that occurs when individuals worry about missing out on wonderful experiences of others or something else (12–14). People with high levels of fear of missing out are more likely to use social media, which makes them more likely to develop mobile phone addiction (15–17). A study by the China Psychological Information Network (CPIN) reported (18) that more than 15% of social media users have experienced a severe fear of missing out; nearly 33% of users check Weibo frequently to avoid missing the latest topics; and 60% of users feel psychologically uneasy if they do not have a smartphone or their smartphone loses power. Individuals undergoing high levels of fear of missing out tend to display updates about themselves or track the lives of others through social media, thus creating irrational expectations about the social features of smartphones and investing more time and energy in smartphone use. Previous researches also demonstrated that individuals who experienced fear of missing out may attempt to reduce their anxiety by keeping on checking information about others through social media (19, 20). These behaviors immensely affect the daily life and study of college students. According to the Self-Determination Theory (SDT), the root cause of individuals’ fear of missing out is the absence of basic psychological needs (21–23). The individual with fear of missing out will be bound to seek specific channels or platforms to meet their basic psychological demands, and the accessibility, as well as convenience of smartphones, make them an ideal social interaction tool. Studies have confirmed that the higher level of an individual’s fear of missing out, the more obvious the behavior of mobile phone addiction (24, 25). Therefore, this study proposes hypothesis 1: Fear of missing out positively predicts mobile phone addiction.

### 1.2 Depression as a mediator

Depression is a kind of morbid psychology defined as a mood disorder by the World Health Organization, whose typical symptoms are primarily being down in spirits, lack of interest, losing the sense of pleasure, feeling exhausted throughout the day, decreasing energy, and so on. Previous studies suggested that individuals with a high degree of fear of missing out experience chronic anxiety because they worry about missing the exciting experiences of others or fail to receive and process the news in time, which can lead to negative emotions such as depression (26, 27). This is consistent with Wortham’s view that fear of missing out is a risk factor for passive emotions such as depression (28). Depression Cognitive Theory (DCT) (29) refers to a portion of individuals who suffer from stressful events, that is, the individual experiencing fear of missing out develops negative cognitive biases so that prevent them from processing information correctly, and these cognitive biases recur as well as are not easily controlled, ultimately resulting in depressive moods. There are plenty of researches on the related influencing factors of depression at home and abroad. However, the potential pathway relationship between fear of missing out and depression among college students still needs to be further explored. In addition, studies have shown that higher levels of depression are associated with mobile phone addiction (30). The Affective Processing Model of Negative Reinforcement (APMNR) also argues for the analysis of motivations for addictive and problematic behaviors from an emotional processing perspective (31). The theory holds that avoidance of negative emotions is the motivation for addictive behaviors, as well as an intrinsic mechanism for the formation, maintenance, and relapse of addictions. When an individual is depressed, one will want to satisfy basic psychological needs by pleasing oneself through online channels, such as the joy of winning a game, or the inner support from the number of views and likes on WeChat or Weibo, and so on. The smartphone is known as a convenient mobile device by the public, which builds a bridge to eliminate negative emotions. Based on the above analysis, this study proposes hypothesis 2: depression mediates the relationship between fear of missing out and mobile phone addiction.

### 1.3 Loneliness as a moderator

DeJong thinks that (32) loneliness is a state of subjective social isolation accompanied by painful feelings of not being accepted due to personal isolation or lack of interactions with others. It has been shown that persistent and greater loneliness is a predisposing factor for depression (33–36). Fear of missing out is associated with mobile phone addiction among college students, but different individuals are not necessarily affected by fear of missing out to the same degree. College students are in a critical period of life development. Since individual development depends on the joint actions of individual and environmental factors (37), they are eager to maintain good interpersonal relationships while learning professional knowledge. However, due to personality, cognition, and other reasons, they have not yet achieved the expected goal, and the accompanying depression as well as frustration make loneliness arise. The Risk Enhancement Model (REM) (38) suggests that one risk factor can enhance the risk of another, so the effect of fear of missing out on mobile phone addiction among college students may be moderated by loneliness.

**Figure 1:**
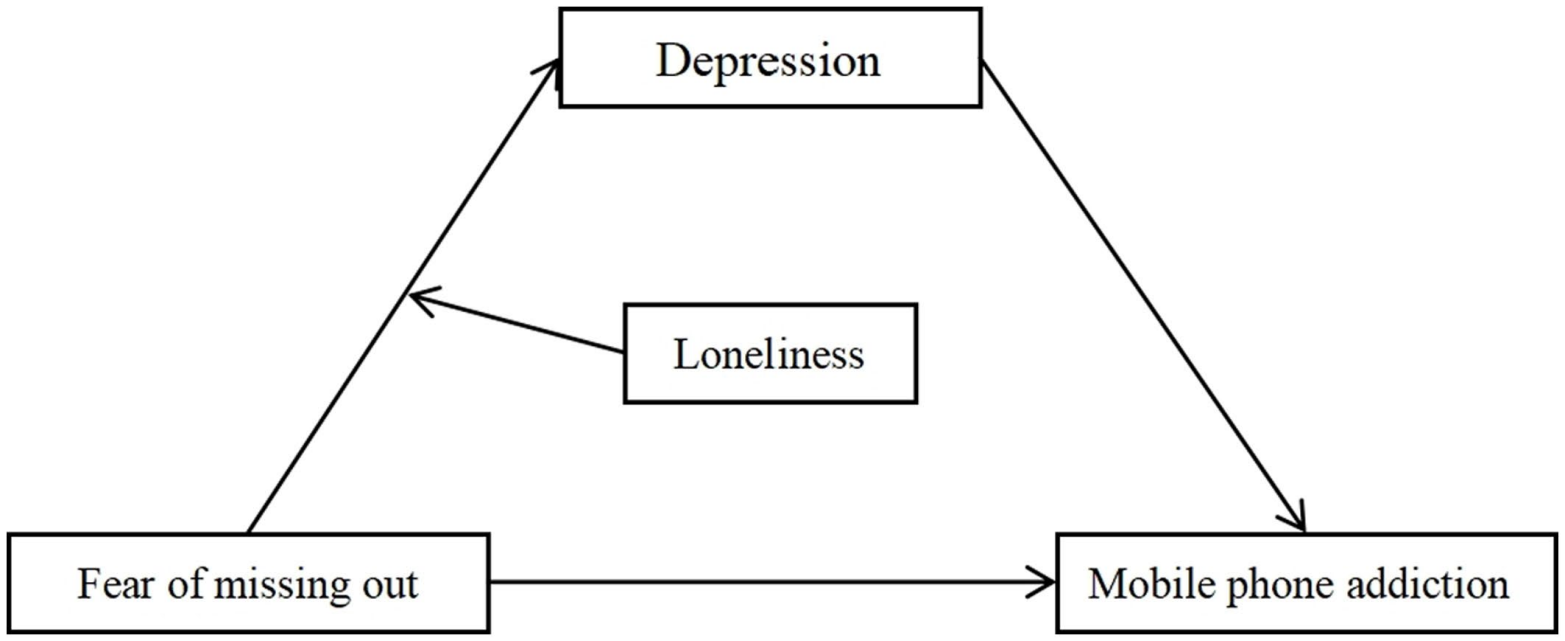
Hypothetical model for this study.

The impact of loneliness on the physical and mental health of the human body can be divided into the following points: First, from the view of physiological mechanism analysis, the theory of excessive serotonin in autism believes that autism arises from excessive exposure of individuals to serotonin in the early stage, which reduces the serotonin receptors, in turn, low serotonin content will lead to depression, emotional impulse, and other negative emotions (39, 40). Secondly, loneliness can also mediate the addictive behavior of individuals. A large number of studies have proved that loneliness is the primary risk of mobile phone addiction, and college students with loneliness are more likely to develop mobile phone addiction (41, 42). For example, to relieve negative emotions and feel the joy brought by social support and interpersonal communication, some individuals may be inclined to choose network social platforms, resulting in their addiction to mobile phone use, which may further develop into mobile phone addiction. Individuals who suffer from fear of missing out are more likely to think that they are not fully integrated into social groups and worry about being unable to take part in social activities timely. In this case, with the participation of loneliness, they may be more inclined to choose the negative coping style, which is to immerse in the immediate pleasure of mobile phone use. In conclusion, this study proposes hypothesis 3: loneliness moderates the relationship between fear of missing out, depression, and mobile phone addiction. By constructing a moderated mediation model (Figure 1), the present study explored the influence of fear of missing out on college students’ mobile phone addiction. At the same time, to verify the mediating role of depression as well as the moderating role of loneliness to provide a theoretical basis and objective empirical evidence for improving college students’ mobile phone addiction.

## 2 Materials and methods

### 2.1 Participants

A convenience sampling method was used to test students at a university in Jilin Province, China, on an online questionnaire platform to obtain a representative sample of college students with mobile phone addiction. According to Kendall’s sample estimation principle, the number of observed samples was 10–20 times the number of items, at the same time, given the possible invalidity of the questionnaire, the planned sample size was increased by 10%. Therefore, the required sample size was at least 715. We distributed 750 questionnaires in electronic forms to the students at our request, and 720 valid questionnaires were obtained (the effective rate of the questionnaires was 96.00%). The ages of the subjects ranged from 17 to 29 years old (*M* = 21.72 years old, SD = 2.43), including 252 (35.0%) were male and 468 (65.0%) were female, 498 (69.2%) were undergraduate students and 222 (31.8%) were graduate students.

### 2.2 Mobile phone addiction

The mobile phone addiction index scale developed by Leung (43) was used to measure the tendency of mobile phone addiction among college students. The scale consists of 17 items, using a 5-point Likert-type scale (from 1 = “never” to 5 = “always”), and the responses are summed up, with a total score ranging from 17 to 85, 17–33 is classified as having no mobile phone addiction, 34–50 is defined as having mild mobile phone addiction, 51–67 suggests moderate mobile phone addiction and the range of 68–85 is regarded as severe mobile phone addiction, and the higher the score, the higher the degree of mobile phone addiction. This scale has shown good reliability and validity among Chinese college students (44–46). In this study, Cronbach’s α coefficient for this scale was 0.937.

### 2.3 Fear of missing out

The fear of missing out scale was designed by Przybylski and translated by Li et al. (12, 47). The applicability test was carried out with a sample of Chinese college students. It was divided into two dimensions: fear of missing information and fear of missing situations. It included 8 items (for example, “I am afraid that other people have more wonderful experiences and gains than me,” etc.), and adopted a 5-point Likert-type scale scoring method (from 1 = “strongly disagree” to 5 = “strongly agree”). The total score is the sum of the scores for each item ranging 8 to 40. Higher scores indicate a higher degree of fear of missing out. The overall reliability and validity index of the scale has proved good among Chinese college students (48). The Cronbach’s α coefficient of this scale was 0.880.

### 2.4 Depression

The depression questionnaire in the Chinese depression-Anxiety-Stress scale (Depression-Anxiety-Stress Scale) revised by Gong et al. (49). was used to measure and evaluate the depression of the subjects. Using the 4-point Likert-type scale, the depression scale score is the sum of the seven items scored multiplied by 2, and ranges from 0 to 42 points, 0–9 proves normal, 10–13 is mild depression, 14–20 is moderate depression, 21–27 is as severe depression, and 28–42 is defined as extremely severe which suggests that the individual is in a negative emotional state, with higher scores indicating higher levels of depression. In this study, Cronbach’s α coefficient of this subscale was 0.902.

### 2.5 Loneliness

This study adopts the USL-8 loneliness scale revised by Hays et al. to measure the extent of individuals’ loneliness (50), which consists of 8 items. In this scale, the 3rd and 6th items were scored in reverse. And the level of loneliness was rated using a 4-point Likert-type scale (1 = “never,” 4 = “always”), with higher total scores indicating higher levels of loneliness. Yan Liu introduced the ULS-8 into China and administered it to college students with good reliability and validity (51). The Cronbach’s α coefficient of the loneliness scale in the present study was 0.817.

### 2.6 Data analysis

In the present study, SPSS and Hayes’ SPSS macro program PROCESS were used for statistical analysis of the data. Descriptive statistical analysis was used to describe the basic sociodemographic characteristics of participants and their scores on study variables. Harman single factor test was used to determine the effect of common method variance on the study results (52). Pearson correlation analysis was used to explore the correlation between fear of missing out, depression, loneliness, and mobile phone addiction. The mediating effects of depression were analyzed using PROSCESS model 4, and then whether the direct effects and mediating effects were moderated was analyzed using PROSCESS model 7 (53).

## 3 Results

### 3.1 Descriptive statistics and the correlation among the studied variables

Table 1 presents the descriptive statistics and Pearson correlation analysis for studied variables, including the mean value, standard deviation, and correlation strength. The results showed that the correlation coefficients among all studied variables ranged from 0.363 to 0.529, and *p* < 0.01 significance level for them. In addition, fear of missing out, depression, loneliness, and mobile phone addiction were positively correlated with each other.

**Table 1:** Descriptive statistics and correlations among some of the observed variables.

Variables	*M*	SD	1	2	3	4
1 Fear of missing out	23.87	6.39	1			
2 Depression	11.13	9.18	0.374**	1		
3 Loneliness	17.75	4.52	0.412**	0.529**	1	
4 Mobile phone addiction	47.32	14.36	0.411**	0.490**	0.363**	1

**p < 0.01.

### 3.2 Testing for the mediating effect of depression

Model 4 in the SPSS macro program compiled by Hayes was used to analyze the mediating role of depression in the influence of fear of missing out on mobile phone addiction. The difference analysis of demographic variables in this study showed that gender and grade had significant effects on mobile phone addiction (*t* = 3.71, *p* < 0.001; *t* = 2.24, *p* < 0.05). Therefore, gender and grade were included in the subsequent analysis as control variables. Table 2 shows the results of this study: First, the fear of missing out significantly positively predicted mobile phone addiction (*β* = 0.92, *p* < 0.001); Secondly, the fear of missing out had a significant positive predictive effect on depression (*β* = 0.54, *p* < 0.001). Finally, when both fear of missing out and depression predicted mobile phone addiction, depression significantly positively predicted mobile phone addiction (*β* = 0.64, *p* < 0.001), and the positive prediction effect of fear of missing out on mobile phone addiction was still significant (*β* = 0.57, *p* < 0.001). However, it indicated that the predictive value of fear of missing out on mobile phone addiction decreased from 0.92 to 0.57, suggesting that depression played a partial mediating role in the influence of fear of missing out on mobile phone addiction. The mediating effect is 0.35, and its 95% Bootstrap confidence interval is [0.26, 0.44], excluding 0. The mediating effect accounts for 37.91% of the total effect. Therefore, the mediating effect of depression between fear of missing out and mobile phone addiction is established.

**Table 2:** Regression results for the mediating effect of depression (mediation model).

Model						
		Fitting index			Coefficient and significance	
		*R*	*R* ^2^	*F*	*β*	*t*
**Model 1: total effect model**						
Outcome variables	Predictor variables					
Mobile phone addiction	Constant	0.433	0.188	55.122	25.350^***^	12.707
	Gender				−2.540^*^	−2.471
	Grade				3.056^**^	2.887
	Fear of missing out				0.918^***^	12.039
**Model 2: mediator variable model**						
Depression	Constant	0.388	0.150	42.279	−1.747	−1.328
	Gender				1.198	1.783
	Grade				−1.434^*^	−2.071
	Fear of missing out				0.540^***^	10.830
**Model 3: dependent variable model**						
Mobile phone addiction	Constant	0.576	0.332	88.710	26.475^***^	14.478
	Gender				−3.311^***^	−3.542
	Grade				3.980^***^	4.129
	Fear of missing out				0.570^***^	7.638
	Depression				0.644^***^	12.414
			***β***	**Boot SE**	**BootLLCI**	**BootULCI**
Total effect of fear of missing out on mobile phone addiction			0.918	0.076	0.769	1.068
Direct effect of fear of missing out on mobile phone addiction			0.571	0.075	0.424	0.717
Indirect effect of depression			0.348	0.047	0.259	0.444

*p< 0.05, **p < 0.01, ***p < 0.001. LL; low limit, CI; confidence interval, UL; upper limit.

### 3.3 Testing for the moderated effect of loneliness

The SPSS macro PROCESS (model 7) was used to verify the moderated mediation model. As can be seen in Table 3, when loneliness was included as a moderator in the regression equation, the interaction term of fear of missing out and loneliness had a significant predictive effect on depression (*β* = 0.03, *p* < 0.01). It suggested that loneliness played a moderating role in the influence of fear of missing out on depression.

**Table 3:** Regression results for the conditional indirect effects (moderated mediation).

Model						
		Fitting index			Coefficient and significance	
		*R*	*R* ^2^	*F*	*β*	*t*
**Model 1: mediator variable model**						
Outcome variables	Predictor variables					
Depression	Constant	0.567	0.322	67.741	10.846^***^	25.486
	Gender				0.793	1.318
	Grade				−1.004	−1.616
	Fear of missing out				0.300^***^	6.047
	Loneliness				0.902^***^	13.083
	Fear of missing out × Loneliness				0.026^**^	2.845
**Conditional direct effect analysis at values of Loneliness (M ± SD)**						
			***β***	**Boot SE**	**BootLLCI**	**BootULCI**
*M* − 1 SD (−4.747)			0.113	0.040	0.037	0.192
*M* (0.253)			0.198	0.041	0.121	0.283
*M* + 1 SD (4.253)			0.265	0.061	0.156	0.392

*p < 0.05, **p < 0.01, ***p < 0.001. LL; low limit, CI; confidence interval, UL; upper limit.

**Figure 2:**
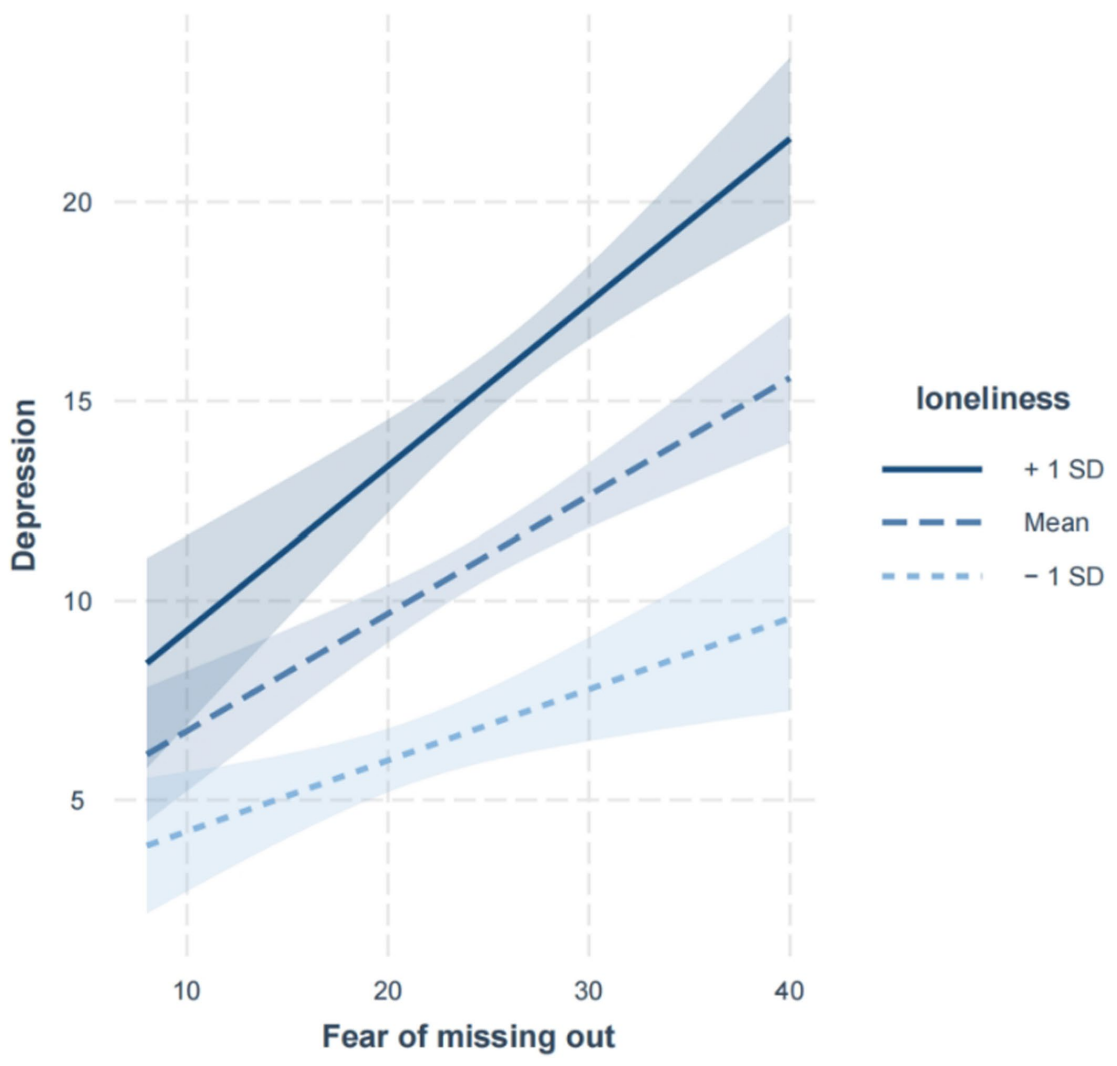
Loneliness moderates the relationship between fear of missing out and depression.

Besides, on this basis, we developed a simple slope analysis to decompose this significant interaction effect (54). The results of a simple slope analysis were shown in Figure 2. As can be seen from Figure 2, the impact of the fear of missing out on depression was positive and significant regardless of their level of loneliness. The difference was that the influence of fear of missing out on depression performed stronger among college students with high levels of loneliness (simple slope = 0.412, *t* = 6.030, *p* < 0.01) than that of college students with low levels of loneliness (simple slope = 0.176, *t* = 2.910, *p* < 0.01).

## 4 Discussion

This study explored the influence of fear of missing out on mobile phone addiction, as well as the mediating role of depression and the moderating role of loneliness, and constructed a moderated mediation model based on previous researches and related theories. Studies indicated that fear of missing out was significantly positively associated with mobile phone addiction, and hypothesis 1 was supported. The higher the level of individuals’ fear of missing out, the higher the degree of mobile phone addiction. Depression partially mediates the relationship between fear of missing out and mobile phone addiction, and hypothesis 2 was supported. In addition, the indirect effect of the fear of missing out on mobile phone addiction was exacerbated when the public experienced more loneliness in their daily lives, and hypothesis 3 was supported. These findings not only helped to identify risk factors of mobile phone addiction behavior, explored the path relationship between mobile phone addiction behavior and related factors, but also guided college students to form a correct attitude and cognitive system of using mobile phones. More important is to improve their physical and mental health, and to provide a theoretical basis for future intervention measures for college students’ mobile phone addiction behavior.

### 4.1 Fear of missing out and mobile phone addiction

The present study found that fear of missing out significantly positively predicted mobile phone addiction, indicating that fear of missing out was a risk factor for mobile phone addiction. Consistent with previous research results (55), the fear of missing out could have an impact on individual behavior and was an important influencing factor for behaviors such as mobile phone addiction. The higher the degree of individuals’ fear of missing out, the more possibly they tend to develop mobile phone addiction. That may be because individuals who are in a state of fear of missing out will consume their self-control and have a stronger desire for the pleasure brought by mobile phones, resulting in individuals being unable to withdraw from the state of using mobile phones promptly (56). At the same time, the fear of missing out due to the lack of timely access to information may stimulate non-adaptive cognition, and individuals believe that the network environment can better meet their needs than reality (57), which makes individuals more addicted to mobile phones. Most previous studies mainly paid more attention to the role of fear of missing out as an intermediary factor in the relationship between mobile phone addiction (58, 59), but this study directly analyzed it as an independent variable to study the direct impact of fear of missing out as an emotional cognition on mobile phone addiction and the related factors affecting the direct path of the both.

### 4.2 Depression as a mediator

The results of the mediation effect test showed that the fear of missing out could not only predict mobile phone addiction through the direct path but also affect mobile phone addiction through the indirect path of depression. Studies have found that fear of missing out can enhance depression, possibly because individuals with a high level of fear of missing out are more afraid of missing external dynamics and social information, thus falling into anxiety, depression, and other negative emotions (26, 27). To change their negative emotional states, individuals often look for external stimuli (such as mobile phones) to release negative emotions or transfer emotional attention. That is consistent with previous research results, that is, users’ Emotional cognitive experience is a direct influencing factor for individuals’ excessive use of mobile phones (12, 60–62), which is also consistent with the idea of the Emotional enhancement Effect (30). A large number of empirical studies (63–65) have shown that depressed individuals are more likely to become dependent on the Internet than other individuals, leading to indulging in mobile phones. To sum up, the fear of missing out can cause depression among college students and further result in the formation of mobile phone addiction.

### 4.3 Loneliness as a moderator

The fear of missing out is related to mobile phone addiction among college students. Still, the indirect predictive effect of fear of missing out on mobile phone addiction is associated with the level of loneliness. To put this into a specific context, the mediating effects of depression in fear of missing out and mobile phone addiction are more obvious in individuals with high levels of loneliness. That may be because loneliness, as a negative emotion or coping style, enhances the direct effect of the fear of missing out on depression and the indirect effect of mobile phone addiction, which is also supported by the risk enhancement model (38).

Loneliness and fear of missing out are both passive emotions and risk factors for depression (34, 41, 42), which can strengthen the effect of fear of missing out on depression. College students, who face the pressure of heavy schoolwork, worry about being disconnected from society, fall into the confusion of interpersonal relationships and the sadness of being separated from their families, feel a high level of loneliness, and may look for online social support through social media to enrich their hearts. The smartphone, a convenient and portable mobile device, has become the best media tool for them to obtain their inner satisfaction and needs through immersion in the pleasure of using mobile phones. This is consistent with the idea of the emotional enhancement effect (30), that is, an individual in a negative mood is more inclined to relieve and suppress it through entertainment and leisure activities. Therefore, people with a high degree of loneliness and fear of missing out have a higher risk of depression and are more likely to develop mobile phone addiction behaviors.

## 5 Limitations and implications

This study provides a theoretical framework for preventing mobile phone addiction among college students. First of all, the application of self-determination theory proves that fear of missing out can significantly positively predict mobile phone addiction. Therefore, to alleviate the fear of missing out among college students, schools can establish a fast information channel and actively organize activities to break the information gap of students, reducing their fear of missing out. Secondly, this study uses the cognitive theory of depression and the negative reinforcement emotion processing model to provide theoretical support for the research on the mediating role of depression between fear of missing out and mobile phone addiction. Fear of missing out can not only directly affect mobile phone addiction, but also indirectly predict through the bridge of depression. Therefore, universities should pay attention to students’ mental health and reduce the risk of depression. Finally, the risk enhancement model is applied to verify the moderating effect of loneliness. Students with high loneliness have a higher level of depression than students with low loneliness, which virtually increases the risk of mobile phone addiction.

Although the present study introduced the mechanism of the influence of fear of missing out on mobile phone addiction and enriched the contents of previous studies on the risk factors of mobile phone addiction, this study also has some limitations. Firstly, the current cross-sectional study was unable to draw rigorous and expected causal relationships. Longitudinal or experimental studies should be conducted to determine the causal direction between fear of missing out, depression, and loneliness. Future studies should also use ingenious intervention design to examine the moderating effect of loneliness by comparing the change in the influence of fear of missing out on depression before and after the intervention. Secondly, because this study was conducted on a group of college students at a university in Jilin Province, the universality of the research results was affected, and the conclusions of this study could not be extended to other social groups, such as teenagers, the older adult, and other populations from different cultures.

## 6 Conclusion

Through a questionnaire survey, this study found that there was a significant positive correlation between the fear of missing out and mobile phone addiction, and depression was a vital medium for the fear of missing out to contribute to mobile phone addiction among college students. At the same time, loneliness is the catalyst of depression induced by fear of missing out, and the indirect influence of fear of missing out on mobile phone addiction was mediated by loneliness. To prevent the mobile phone addiction behavior of college students, we need to pay more attention to improving their mental health and enhancing their emotional regulation ability.

## Data availability statement

The datasets presented in this article are not readily available because this data is only applicable to this research group. Requests to access the datasets should be directed to NL, L19981224525@163.com.

## Ethics statement

The studies involving humans were approved by School of Public Health of Jilin University. The studies were conducted in accordance with the local legislation and institutional requirements. The participants provided their written informed consent to participate in this study.

## Author contributions

NL: Writing – review & editing, Writing – original draft, Validation, Supervision, Software, Project administration, Methodology, Investigation, Formal analysis, Data curation, Conceptualization. SZ: Writing – review & editing, Supervision, Software, Methodology, Investigation, Data curation, Conceptualization. WZ: Writing – review & editing, Supervision, Methodology, Data curation. YS: Writing – review & editing, Supervision, Methodology, Data curation. XZ: Writing – review & editing, Conceptualization.
